# The lnc-CTSLP8 upregulates CTSL1 as a competitive endogenous RNA and promotes ovarian cancer metastasis

**DOI:** 10.1186/s13046-021-01957-z

**Published:** 2021-05-01

**Authors:** Xinjing Wang, Xiaoduan Li, Feikai Lin, Huizhen Sun, Yingying Lin, Ziliang Wang, Xipeng Wang

**Affiliations:** 1 Department of Gynecology and Obstetrics, XinHua Hospital, Shanghai JiaoTong University School of Medicine, 1665 Kongjiang Rd, Yangpu District, Shanghai, 200092 China; 2 Department of Gynecology, Shanghai First Maternity and Infant Hospital, Tongji University School of Medicine, Shanghai, China; 3 Department of Neurosurgery, Renji Hospital, Shanghai JiaoTong University School of Medicine, Shanghai, China

**Keywords:** Lnc-CTSLP8, Ovarian cancer, ceRNA, CTSL1, Metastasis

## Abstract

**Background:**

Ovarian cancer is highly lethal and has a poor prognosis due to metastasis. Long non-coding RNAs (lncRNAs) are key regulators of tumor development, but their role in ovarian cancer metastasis remains unclear.

**Methods:**

The expression of lnc-CTSLP8 in ovarian cancer was analyzed in public databases (TCGA and GEO) and validated via qRT-PCR. Lnc-CTSLP8 overexpression and knockout cell lines were constructed using a lentiviral vector and the CRISP/Cas9 system. Cell proliferation, colony formation, migration, and invasion were analyzed. An ovarian orthotopic tumor mouse model was used for the in vivo study. Changes in autophagosomes, autolysosomes, and mitochondria in ovarian cancer cells were observed via transmission electron microscopy. EMT markers were detected by immunoblotting and immunofluorescence assays. RNA immunoprecipitation, RNA pull-down, and dual luciferase reporter assays were performed to confirm the interaction between lnc-CTSLP8 and miR-199a-5p.

**Results:**

A novel pseudogene, lnc-CTSLP8, was identified in ovarian cancer, with significantly elevated expression in metastatic tumor tissues compared to primary ovarian tumors. When overexpressed, lnc-CTSLP8 promoted ovarian cancer in vitro and in vivo by acting as a sponge for miR-199a-5p. Autophagy and EMT in ovarian cancer were also enhanced by lnc-CTSLP8. Mechanistically, lnc-CTSLP8 upregulated CTSL1 as a competitive endogenous RNA and exhibited oncogenic effects. Moreover, CTSL1 inhibitor treatment and miR-199a-5p overexpression abrogated the effects of lnc-CTSLP8 overexpression.

**Conclusions:**

lnc-CTSLP8 acts as a ceRNA in ovarian cancer and represents a potential therapeutic target for metastatic ovarian cancer.

**Supplementary Information:**

The online version contains supplementary material available at 10.1186/s13046-021-01957-z.

## Background

Ovarian cancer is the fifth leading cancer-related cause of death in women, with the highest mortality rate among gynecological malignancies [1]. In China, over 52,000 new ovarian cancer patients are diagnosed, and 22,500 patients die from ovarian cancer each year [2]. Early diagnosis is difficult due to the lack of established biomarkers and specific clinical symptoms, leading to extremely poor prognosis [3, 4]. More than 50% of patients are diagnosed at an advanced stage with widespread metastasis [5]. Therefore, novel biomarkers for early diagnosis and therapeutic targets for ovarian cancer metastasis are of urgent necessity.

Non-coding RNAs (ncRNAs) play an important role in various diseases, including ovarian cancer [6, 7]. Our previous study showed that distinct transcriptome patterns of monocytes, including the ncRNA expression profile, were potentially associated with tumor angiogenesis in ovarian cancer [8]. In addition, ncRNAs such as miRNAs could be transferred via exosomes and suppress T-cell differentiation within the ovarian cancer microenvironment, leading to tumor progression [9]. Recently, intricate interactions among different RNA types have been reported and described as competing endogenous RNA (ceRNA) networks, which include mRNAs and non-coding RNAs such as long non-coding RNAs (lncRNAs) [10, 11]. Further, lncRNAs have been shown to play important roles in tumorigenesis and metastasis through their function as ceRNAs [12, 13].

Pseudogenes are a subclass of lncRNAs that share high sequence homology with protein-coding genes without the ability to encode proteins. Importantly, pseudogenes participate in the post-transcriptional regulation of their protein-coding counterparts [14, 15]. For example, PTEN pseudogene 1 (PTENP1) acts as a decoy against PTEN-targeting miRNAs with tumor suppressor activity [16]. Similarly, BRAF pseudogene 1 (BRAF1) upregulated its cognate BRAF by competitively interacting with shared miRNAs, promoting lymphoma [17]. However, pseudogenes in ovarian cancer are yet to be investigated, which might lead to a better understanding of the role of non-coding RNAs in the disease.

Cysteine cathepsin L (CTSL1) is an important member of the cathepsin family and contributes to tumor progression through different mechanisms, such as the induction protein degradation and autophagy [18]. Moreover, CTSL1 enhances the epithelial-to-mesenchymal transition (EMT) and promotes cancer invasion and metastasis [19, 20]. In ovarian cancer, CTSL1 has been reported as an independent diagnostic and prognostic factor [21]. Moreover, elevated CTSL1 expression was associated with chemoresistance and metastasis of ovarian cancer [22].

In this study, we screened RNA expression patterns in metastatic ovarian cancer tissues via RNA sequencing and identified non-coding CTSL pseudogene 8 (lnc-CTSLP8), which was significantly upregulated. We then established lnc-CTSLP8 overexpression and knockout cell lines to investigate the role of lnc-CTSLP8 in ovarian cancer cells in vitro and in vivo. Mechanistically, we found that lnc-CTSLP8 upregulated its cognate gene CTSL1 by acting as a sponge for miR-199a-5p, and CTSL1 inhibition abrogated the oncogenic effects of lnc-CTSLP8.

## Methods

### Patients and specimens

A total of 76 benign ovarian cyst tissues, 219 epithelial ovarian cancer tissues, and 126 peritoneal metastasis ovarian cancer tissues, together with 38 serum and 38 ascites samples from the same cohort of cancer patients, were collected from benign ovarian cyst or epithelial ovarian cancer patients between 2008 and 2018, after informed consent had been obtained. The histopathological diagnosis, stage, and grade of ovarian cancer were based on the FIGO classification. Overall survival (OS) was defined as the time from the initiation of surgery to death of any cause or the most recent follow-up. The clinical data of ovarian cancer patients are shown in Table S1. This study was approved by the Ethics Committee of the Xinhua Hospital.

### TCGA and GEO data analysis

The lncRNA-seq data were obtained from the Cancer Genome Atlas (TCGA) ovarian cancer cohort of 379 ovarian serous cystadenocarcinoma patients. The expression of lnc-CTSLP8 was normalized by variance-stabilizing transformation (VST) and compared between patients of different stage and grade. The microarray data derived from dataset GSE19829 (https://www.ncbi.nlm.nih.gov/geo/query/acc.cgi?acc=GSE19829) of 28 epithelial ovarian cancer patients were used to analyze the correlation between CTSL1 and lnc-CTSLP8 expression.

### Immunohistochemistry (IHC)

Tissue microarrays (TMAs) were conducted using benign ovarian cyst tissues, epithelial ovarian cancer tissues, as well as peritoneal metastasis ovarian cancer tissues and a CTSL1 antibody (ab203028, Abcam, 1:100 dilution). The intensity of staining was classified as weak, moderate, and strong. The IHC score was calculated as ∑ (PI × I) = (percentage of cells of weak intensity × 1) + (percentage of cells of moderate intensity × 2) + (percentage of cells of strong intensity × 3).

### Elisa

Soluble CTSL1 protein levels in the serum and ascites of ovarian cancer patients were detected via ELISA using the Human Cathepsin L DuoSet ELISA Kit (R&D Systems, USA).

### Cell culture

Human ovarian cancer cell lines were purchased from the Cell Bank of the Chinese Academy of Sciences and cultured in RPMI-1640 medium (Gibco, USA) supplemented with 10% fetal bovine serum (FBS, Gibco, USA) and 1% antibiotics. SKOV3, OVCA420, and Hey cell lines were transfected with a luciferase reporter vector containing a neomycin resistance gene and cultured in complete medium supplemented with 50 μg/ml neomycin. All cell lines were authenticated using short tandem repeat (STR) analysis.

### Lnc-CTSLP8 overexpression and knockout cell lines

The human wild-type lnc-CTSLP8 lentiviral vector was designed by Genomeditech (China) and used to infect SKOV3 and OVCA420 cell lines in order to generate lnc-CTSLP8 overexpression cell lines SKOV3-CTSLP8-OE and OVCA420-CTSLP8-OE, respectively. Negative control cell lines were generated via infection with control lentivirus containing a random sequence (control vectors), and wild-type cells were used as blank controls.

To knock out lnc-CTSLP8 in ovarian cancer cells, eight sgRNAs were designed (Fig. S1B), and the sgRNA sequences are listed in Table S2. After infection and testing, sgRNA2 and sgRNA6 were selected, and a two-gRNA-expressing plasmid was constructed for further lnc-CTSLP8 knockout (Fig. S1C). The Hey cell line was infected with lentiCas9-Blasticidin lentivirus and selected with blasticidin (10 μg/ml). Surviving cells were infected with the H_CTSLP8 (CTSL1P8) sgRNA (PGMLV-GM1) lentivirus and selected with puromycin (2 μg/ml). After selection, the cells were maintained with blasticidin (5 μg/ml) and puromycin (1 μg/ml). The isolated single clone was subjected to PCR and DNA sequencing for knockout validation. Sanger sequencing confirmed the deletion of CTSLP8 in the knockout single clone (Fig. S1D). Sanger sequencing data are provided as supplementary material (16305_T01C-14798CF1_10778-P190923108A_H01.ab1).

The Hey-CTSLP8-KO cell line was used for further experiments. The negative control cell line was generated via infection of Hey cells with control lentivirus containing a random sequence that did not target any genes (control vector), and wild-type Hey cells were used as blank controls.

### RNA sequencing

Ovarian cancer cell lines (SKOV3-CTSLP8-OE and negative controls) were used for RNA sequencing. Total RNA was extracted using the miRNeasy Micro Kit (Qiagen, Germany). Next-generation libraries were then prepared using the VAHTS mRNA-seq v2 Library Prep Kit or the VAHTS TM Total RNA-seq Library Prep Kit (Vazyme, China). Thereafter, RNA-seq libraries were sequenced using the HiSeq X10 system (Illumina, USA). Significantly differentially expressed genes were defined as those with a fold-change > 2 and *p* < 0.05. Gene Set Enrichment Analysis (GSEA) was used for gene function annotation.

### Single-cell RNA-sequencing

Fresh ovarian cancer tissue, adjacent ovarian tissue, and peritoneal metastasis tissue samples were cut into small pieces and then enzymatically digested using the MACS tumor dissociation kit (Miltenyi Biotec, Germany). The cells were filtered using a 70-mm Cell-Strainer (BD Biosciences, USA) and centrifuged at 1200 rpm for 5 min. The pelleted cells were suspended in red blood cell lysis buffer (Miltenyi Biotec, Germany) in order to lyse red blood cells. For droplet-based single-cell RNA sequencing, single cells were processed using the GemCode Gel Bead, Chip, and Library Kit (10x Genomics, USA). A total of 6000 cells were loaded into each sample. Libraries were sequenced using the NovaSeq 6000 System. Single-cell RNA sequencing data were aligned and quantified using Cell Ranger Single-Cell Software Suite 2.3 (10x Genomics, USA).

### Quantitative real-time PCR

Total RNA was extracted from cells or tissues with TRIzol (Invitrogen, USA) and subjected to PCR using the PrimeScript RT-PCR kit (Takara, Japan). The primers for lncRNAs and mRNAs are listed in Table S3. The miRNA was reverse-transcribed into cDNA using the miScript II RT Kit (Qiagen, Germany) and subjected to PCR using the miScript SYBR Green PCR Kit (Qiagen, Germany). The miRNA primers for has-miR-199a-5p were purchased from GenePharma (Shanghai, China). Relative gene expression was calculated using the comparative threshold cycle method.

### Immunofluorescence assay (IF)

Cells or tissues were fixed with 4% paraformaldehyde and permeabilized with 0.3% Triton X-100. Next, the sections were incubated with 5% goat serum (Life Technologies, USA) for 1 h at room temperature, followed by incubation with primary and secondary antibodies. After staining with DAPI (Life Technologies, USA), the sections were imaged under a Leica SP5 confocal fluorescence microscope. The primary antibodies used are listed in Table S4.

### Western blotting

Tissues or cells were lysed in RIPA buffer, and lysates were separated via 10% SDS-PAGE and transferred onto polyvinylidene fluoride membranes (Millipore, USA). The membranes were blocked with 5% BSA for 2 h, incubated with the primary antibodies listed in Table S4, and then incubated with secondary HRP-conjugated antibodies at room temperature for 1 h. A chemiluminescent substrate (Millipore, US) was used to visualize the signals.

### Luciferase assay

HEK293T cells were co-transfected with 100 ng of CTSL1–3’UTR-Luciferase/mut CTSL1–3’UTR-Luciferase plasmids or CTSLP8-Luciferase/mut CTSLP8-Luciferase and predicted miRNAs/miR-NC (negative control), and 20 ng Renilla plasmid was co-transfected to determine relative luciferase activity as the ratio of Firefly luciferase activity to Renilla luciferase activity. Luciferase activity was measured using a dual luciferase reporter system (Promega, USA) 48 h after transfection.

### RNA pull-down assay

Biotinylated miR-199a-5p (5′-CCCAGUGUUCAGACUACCUGUUC-3′) or its mutant (5′-CUGUGACCUCAGACUACCUGUUC-3′) were transfected into SKOV3-CTSLP8-OE cells. The cells were harvested, lysed, sonicated, and incubated with C^− 1^ magnetic beads (Thermo Fisher Scientific, USA) at 4 °C for 3 h. The RNA mix bound to the beads was then eluted and extracted with TRIzol (Invitrogen, USA) for qRT-PCR.

### RNA immunoprecipitation assay

An RNA immunoprecipitation assay (RIP) was carried out in SKOV3 cells using a Magna RIP RNA-Binding Protein Immunoprecipitation Kit (Millipore, USA) according to the manufacturer’s instructions. Argonaute2 (AGO2) antibody (Abcam, cat. #ab186733) and immunoglobulin G (IgG) antibody (Millipore, USA) were used for the RIP assays, and the purified RNAs were extracted and analyzed via qRT-PCR. The primers used are shown in Table S3.

### CCK8 assay

Ovarian cancer cells were seeded into 96-well plates (1500 cells/well), and CCK8 solution (Beyotime, China) was then added to each well daily for a total of 5 days. After incubation for 2 h at 37 °C, the absorbance at 450 nm was measured using a microplate reader (Thermo Labsystems, Finland). Cathepsin L Inhibitor Z-FY-DMK (Sigma, USA) was dissolved in DMSO at 20 μmol/L and added to the medium to obtain a final concentration of 1 μmol/L. The cells were treated with Z-FY-DMK for a total of 5 days. After incubation with CCK8 solution, the absorption at 450 nm was measured. DMSO was used as the negative control.

### Colony formation assay

Ovarian cancer cells were seeded in 6-well plates at 500 cells/well and cultured for 10 days. The colonies were stained with 0.2% crystal violet for 10 min and then counted.

### Migration and invasion assay

A total of 6 × 10^4^ cells were suspended in serum-free DMEM and added to the upper chamber of a Transwell chamber with 8-μm pores (Corning, USA) (for migration assays) or a chamber pre-coated with Matrigel (BD Biosciences, USA) (for invasion assays). DMEM supplemented with 10% FBS was added to the lower chamber. After 24 h, the cells that migrated or invaded the lower chamber were stained with 0.2% crystal violet and counted.

### Transmission electron microscopy (TEM)

Ovarian cancer cells were fixed in fixing solution (Servicebio, China) at 4 °C for 4 h and post-fixed with 1% OsO4 for 2 h at room temperature, followed by dehydration and infiltration. The cells were then embedded, cut into ultrathin sections (60–80 nm), and observed under a transmission electron microscope (HITACHI, Japan).

### Animal tumor model

Animal experiments were approved by the Animal Care and Use Committee of Xinhua Hospital. Female athymic nude mice (6 weeks old) were purchased from Slac Laboratory Animal Center (Shanghai, China) and randomly divided into different groups (*n* = 5) for ovarian orthotopic injection with 5 × 10^5^ SKOV3-CTSLP8-OE, OVCA420-CTSLP8-OE, Hey-CTSLP8-KO cells, or control vectors and wild-type cell lines as negative and blank controls, respectively. Ovarian cancer cells in 10 μL serum-free RPMI-1640 were injected into the left ovarian parenchyma of nude mice. In addition, Z-FY-DMK (100 mg/kg) was intravenously injected through the tail vein each week, and DMSO was used as a negative control.

Two weeks after ovarian orthotopic injection, the tumor-bearing mice were examined for luciferin expression using D-luciferin (100 mg/kg, Invitrogen, US) to assess tumor development every week. After 6 weeks, tumors in each group were harvested following euthanasia, and the weights of tumors and metastasis organs were recorded. For survival analysis, 12 mice from each group were used to calculate OS from the ovarian orthotopic injection of different cell lines to death or to 12 weeks after ovarian orthotopic injection.

### RNA fluorescence in situ hybridization (FISH)

To detect lnc-CTSLP8 expression in ovarian cancer cells and tissues, a lnc-CTSLP8 probe (5′-CY3-GGTTTTAACCTGATCCTTCACAGGACTCAT-3′) was designed and synthesized. The probe signal was detected using a FISH Kit (Servicebio, China) as per the manufacturer’s instructions.

### Statistical analysis

Data are presented as mean ± SEM and were analyzed using SPSS 20.0 software (SPSS Inc., Chicago, IL, USA). Student’s *t*-test, two-way ANOVA, and chi-square tests were used for comparison. Overall survival (OS) was calculated using the Kaplan-Meier method. Statistical significance was set at *p* < 0.05.

## Results

### High expression of CTSL pseudogene 8 in ovarian cancer

**Fig. 1:**
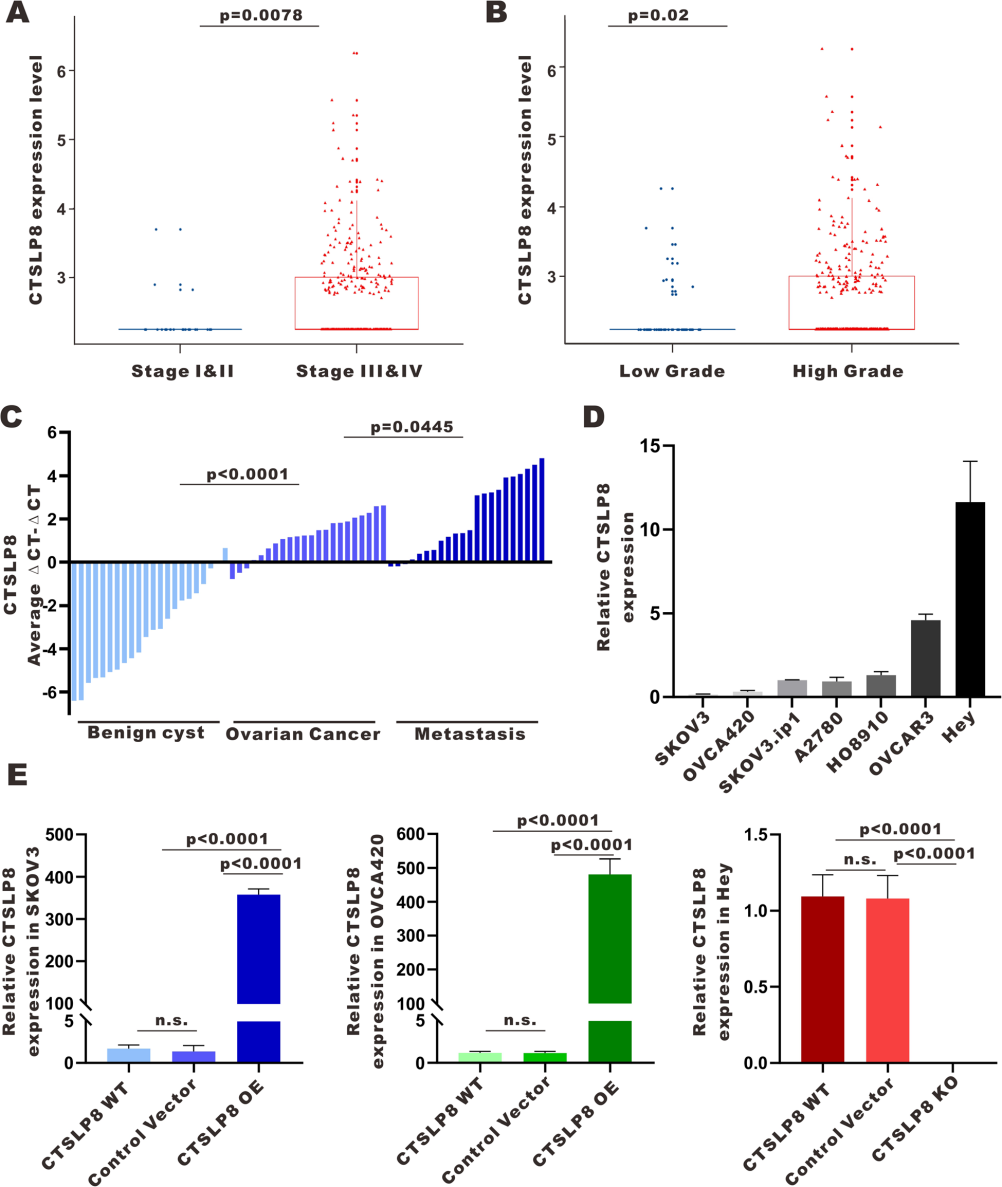
Lnc-CTSLP8 was upregulated in ovarian cancer. **a, b** TCGA data analysis of lnc-CTSLP8 expression in samples from ovarian cancer patients with different disease stages (**a**) and grades (**b**). **c** qRT-PCR of lnc-CTSLP8 expression in benign ovarian cyst tissues (used as control), ovarian cancer tissues, and matched peritoneal metastasis tissues. **d** qRT-PCR of lnc-CTSLP8 expression in different ovarian cancer cell lines. **e** qRT-PCR of lnc-CTSLP8 in CTSLP8 overexpression cell lines (SKOV3-CTSLP8-OE and OVCA420-CTSLP8-OE), the lnc-CTSLP8 knockout cell line (Hey-CTSLP8-KO), negative control cell lines (control vector), and wild-type (WT) cell lines. Data are shown as the mean ± SEM

Based on lncRNA-seq data derived from the TCGA ovarian cancer cohort of 379 patients, lnc-CTLSP8 was significantly elevated in advanced-stage and high-grade ovarian cancer samples (Fig. 1a, b). In addition, through qRT-PCR, we found that lnc-CTSLP8 expression was significantly higher in ovarian cancer tumor tissues (*n* = 22) than in benign ovarian cyst tissues (*n* = 22). Further, lnc-CTSLP8 expression in ovarian cancer peritoneal metastasis tissues (*n* = 22) was even higher than that in ovarian cancer in situ tumor tissues (Fig. 1c).

### Lnc-CTSLP8 promotes the proliferation, migration, and invasion of ovarian cancer cells

To assess the role of lnc-CTSLP8 in ovarian cancer, lnc-CTSLP8 expression in different ovarian cancer cell lines was assessed via qRT-PCR (Fig. 1d). Thereafter, lnc-CTSLP8 was overexpressed in ovarian cancer cell lines with relatively low lnc-CTSLP8 expression (SKOV3 and OVCA420) and knocked out in the ovarian cancer cell line with the highest lnc-CTSLP8 expression (Hey). We analyzed lnc-CTSLP8 expression in lnc-CTSLP8 overexpression/knockout (OE/KO) cell lines relative to the negative control (NC) cell lines (cell lines transfected with control vector) and blank control cell lines (wild-type cells) (Fig. 1e). We also confirmed lnc-CTSLP8 expression in lnc-CTSLP8 overexpression/ knockout cell lines via immunofluorescence assay using an lnc-CTSLP8 RNA probe (Fig. S1A).

**Fig. 2:**
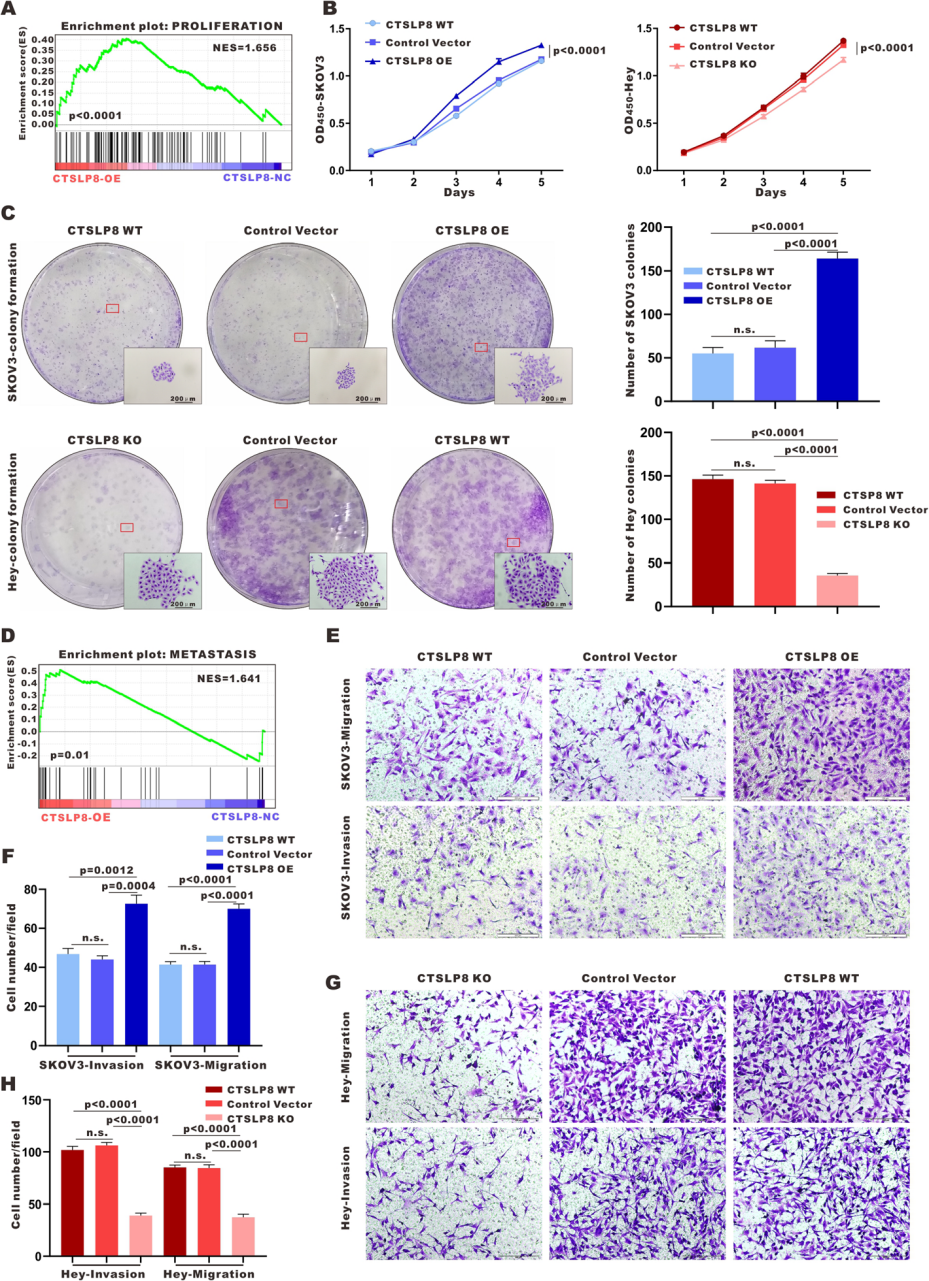
Lnc-CTSLP8 promoted the proliferation and invasion of ovarian cancer cells. **a** and **d** GSEA analysis of SKOV3-CTSLP8-OE and SKOV3-CTSLP8-NC (control vector). The signature was defined by genes with significant expression changes. **b** Cell viability assay and (**c**) colony formation assay in different cell lines. **e-g)** Invasion and migration assays in different cell lines

To explore the function of lnc-CTSLP8 in ovarian cancer, RNA sequencing and Gene Set Enrichment Analysis (GSEA) were conducted in SKOV3-CTSLP8-OE/NC cell lines, highlighting cell proliferation and metastasis as important lnc-CTSLP8-associated pathways in ovarian cancer (Fig. 2a, d). Indeed, the CCK-8 assay revealed that lnc-CTSLP8 overexpression significantly promoted SKOV3 and OVCA420 cell proliferation (Fig. 2b, Fig. S2A), while lnc-CTSLP8 knockout significantly inhibited Hey cell proliferation (Fig. 2b). Colony formation assays indicated that lnc-CTSLP8 overexpression increased the number and size of colonies in SKOV3 and OVCA420 cells (Fig. 2c, Fig. S2B, C). In contrast, lnc-CTSLP8 knockout significantly reduced the number and size of Hey cell colonies (Fig. 2c). Transwell assays revealed that cell migration and invasion were enhanced in lnc-CTSLP8 overexpression SKOV3 and OVCA420 cells (Fig. 2e,f, Fig. S2D, E), while the cell migration and invasion were attenuated after lnc-CTSLP8 knockout in Hey cells (Fig. 2g,h).

### Lnc-CTSLP8 promotes autophagy in ovarian cancer cells

**Fig. 3:**
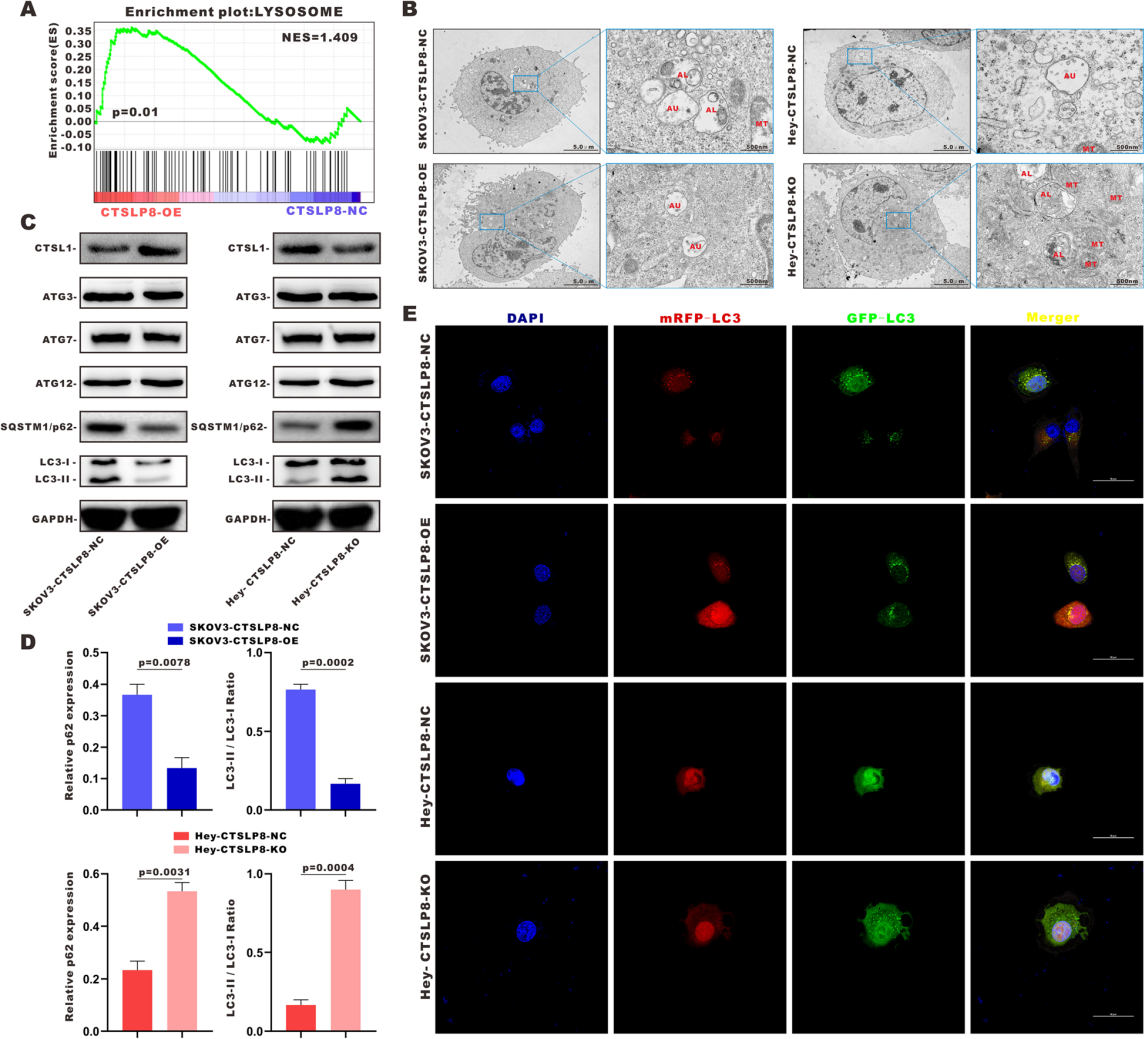
Lnc-CTSLP8 promoted autophagy in ovarian cancer cells. **a** GSEA analysis of SKOV3-CTSLP8-OE and SKOV3-CTSLP8-NC (control vector). The signature was defined by genes with significant expression changes. **b** Transmission electron microscopy (TEM) was used to observe autophagosomes (AU), autolysosomes (AL), and mitochondria (MT) in different cell lines. **c** Immunoblotting of autophagy-related genes in different cell lines. **d** Quantification of immunoblotting analysis revealed the relative SQSTM1-p62 expression and LC3-II/ LC3-I ratio in different cell lines (Student t-test). **e** Immunofluorescence assay of mRFP-GFP-LC3 in different cell lines

GSEA suggested that autophagy was enhanced in ovarian cancer cells under lnc-CTSLP8 overexpression (Fig. 3a). TEM revealed greater numbers of enlarged autophagosomes (AU), autolysosomes (AL), and dysfunctional mitochondria (MT) following lnc-CTSLP8 knockout, whereas overexpression decreased the number of autolysosomes and dysfunctional mitochondria in ovarian cancer cells (Fig. 3b). We also found that LC3 was downregulated in lnc-CTSLP8 overexpression cells while upregulated in their knockout counterparts (Fig. S3A).

Notably, lnc-CTSLP8 overexpression/knockout had a significant effect on the expression of autophagy-specific substrate SQSTM1/p62, but not on that of molecules crucial for autophagosome formation such as autophagy-related 3 (ATG3), ATG7, and ATG12. Meanwhile, ectopic expression of lnc-CTSLP8 significantly altered the LC3-II/LC3-I ratio in ovarian cancer cells (Fig. 3c, d). mRFP-GFP-LC3 expression was then detected via immunofluorescence (Fig. 3e). Since GFP immunofluorescence could be compromised by the acidic environment within the autolysosome, we used mRFP-GFP-LC3 as an index of autophagy flux in ovarian cancer cells. Immunofluorescence assays indicated that autophagy flux was enhanced by lnc-CTSLP8 overexpression and suppressed by lnc-CTSLP8 knockout.

### Lnc-CTSLP8 promotes EMT in ovarian cancer cells

**Fig. 4:**
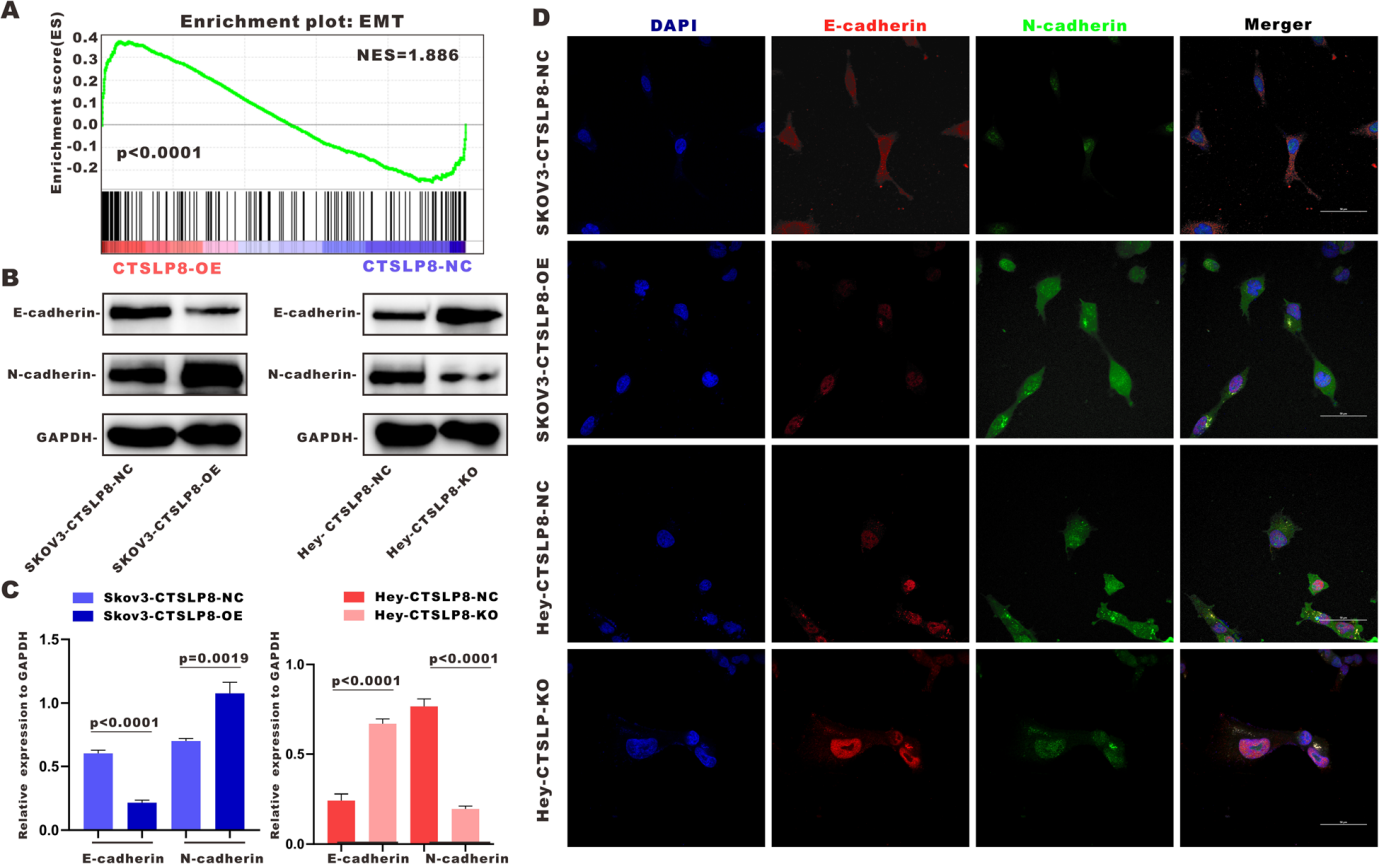
Lnc-CTSLP8 promoted EMT in ovarian cancer. **a** GSEA analysis of SKOV3-CTSLP8-OE and SKOV3-CTSLP8-NC (control vector) cells. The signature was defined by genes with significant expression changes. **b** Immunoblotting of EMT markers in different cell lines. **c** Quantification of immunoblotting analysis showed relative EMT marker expression in different cell lines (Student’s t-test). **d** Immunofluorescence assay of E-cadherin (red) and N-cadherin (green) in different cell lines

GSEA suggested that the EMT was promoted in ovarian cancer cells after lnc-CTSLP8 overexpression (Fig. 4a). Immunoblotting of EMT markers E-cadherin and N-cadherin in SKOV3-CTSLP8-OE and Hey-CTSLP8-KO cell lines indicated that the EMT was indeed promoted via lnc-CTSLP8 overexpression and suppressed under lnc-CTSLP8 knockout (Fig. 4b, c). Immunofluorescence staining of E-cadherin and N-cadherin revealed similar results (Fig. 4d). In addition, the expression of ZEB1 and Snail, key regulators of the EMT, was detected via immunofluorescence staining (Fig. S3B). Overexpression of lnc-CTSLP8 upregulated while lnc-CTSLP8 knockout suppressed the expression of both regulators.

### Lnc-CTSLP8 promotes the tumorigenesis and metastasis of ovarian cancer in vivo

**Fig. 5:**
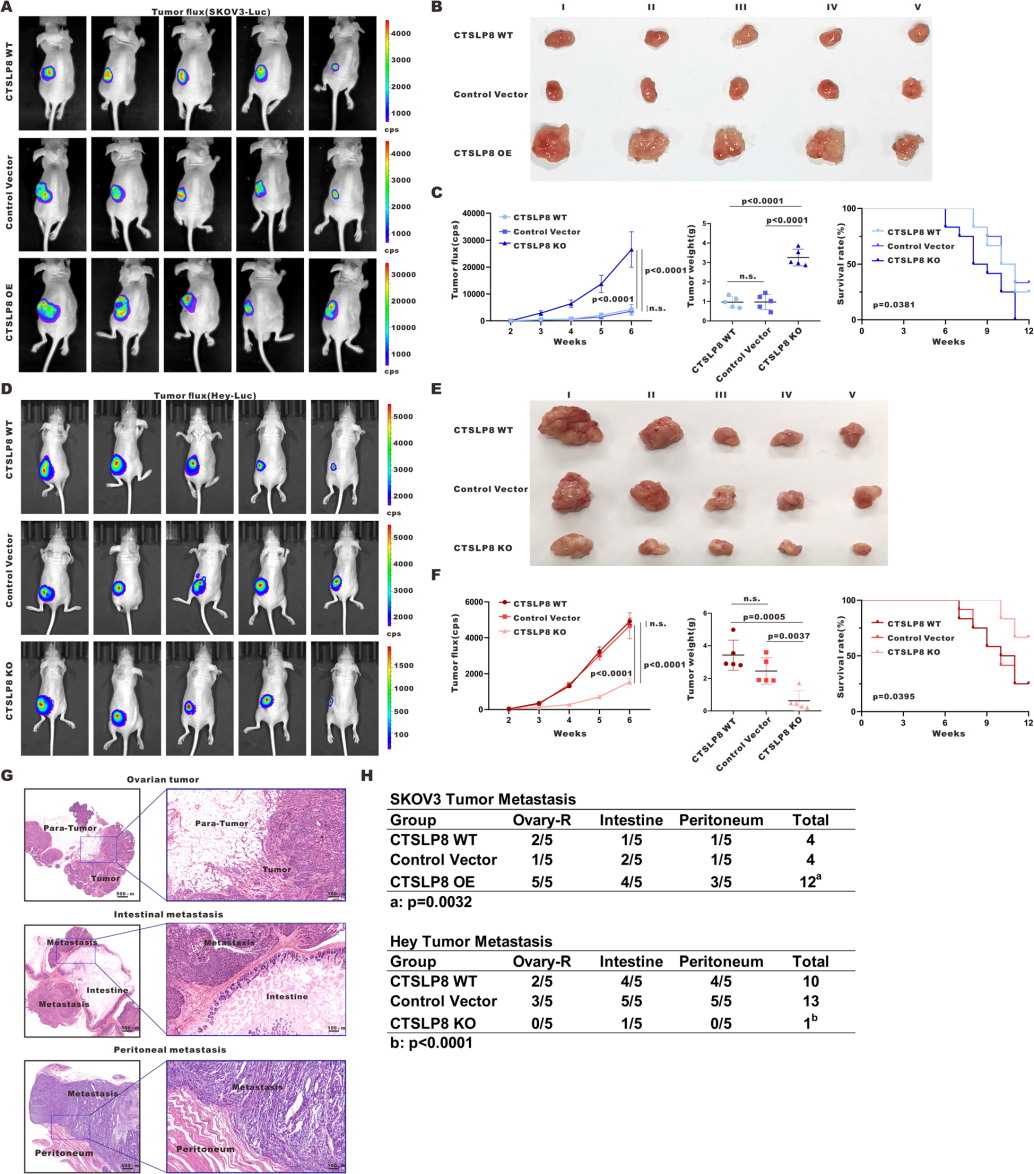
Lnc-CTSLP8 promoted tumor growth and metastasis of ovarian cancer in vivo. **a** and **d** Representative bioluminescence image of ovarian tumor mouse models for the detection of tumor growth in different groups. **b** and **e** Size of tumor-bearing nude mice in different groups. **c** and **f** Quantification of tumor fluorescence intensity, tumor weight, and mouse survival in different groups. **g** HE staining of ovarian tumor and metastasis tissues. **h** Metastases counts in different groups

To validate the function of lnc-CTSLP8 in vivo, we established an orthotopic ovarian cancer mouse model. As shown in Fig. 5a and d, lnc-CTSLP8 overexpression promoted the growth of SKOV3 cell-derived tumors, while lnc-CTSLP8 depletion led to decreased growth of Hey cell-derived tumors. Lnc-CTSLP8 overexpression also increased the volume and weight of SKOV3 cell-derived tumors, while lnc-CTSLP8 knockout had the opposite effect in Hey cell-derived tumors (Fig. 5b, e). Moreover, lnc-CTSLP8 overexpression led to reduced overall survival in tumor-bearing mice, whereas mice with Hey-CTSLP8-KO tumors had longer overall survival than their Hey-CTSLP8-NC and Hey-CTSLP8-WT counterparts (Fig. 5c, f). Metastasis tissues in the right ovary, intestine, and peritoneum of tumor-bearing mice were harvested for HE staining (Fig. 5g). SKOV3-CTSLP8-OE-inoculated mice developed more metastases, while less metastases were observed in Hey-CTSLP8-KO mouse tumor models (Fig. 5h).

An orthotopic ovarian cancer model was generated using OVCA420-CTSLP8-OE and associated control cell lines. In agreement with previous results, lnc-CTSLP overexpression promoted OVCA420 tumor growth, volume, weight, and metastases in mice. Further, OVCA420-CTSLP8-OE-inoculated mice had significantly reduced overall survival compared to control groups (Fig. S2F-J).

The overexpression/knockout of lnc-CTSLP8 in mouse orthotopic ovarian tumor tissues was confirmed via immunofluorescence using the lnc-CTSLP8 probe (Fig. S4A). The expression of LC3, SQSTM1/p62, E-cadherin, and N-cadherin was detected via immunofluorescence assays (Fig. S4B, C). Reduced LC3 and SQSTM1/p62 were observed in SKOV3-CTSLP8-OE ovarian tumor tissues. Further, lnc-CTSLP8 overexpression upregulated N-cadherin, indicating that lnc-CTSLP8 might promote ovarian cancer autophagy and EMT in vivo. In contrast, we observed suppressed autophagy and EMT in the ovarian tumor tissues of Hey-CTSLP8-KO mice.

### Lnc-CTSLP8 acts as a sponge for miR-199a-5p in ovarian cancer

**Fig. 6:**
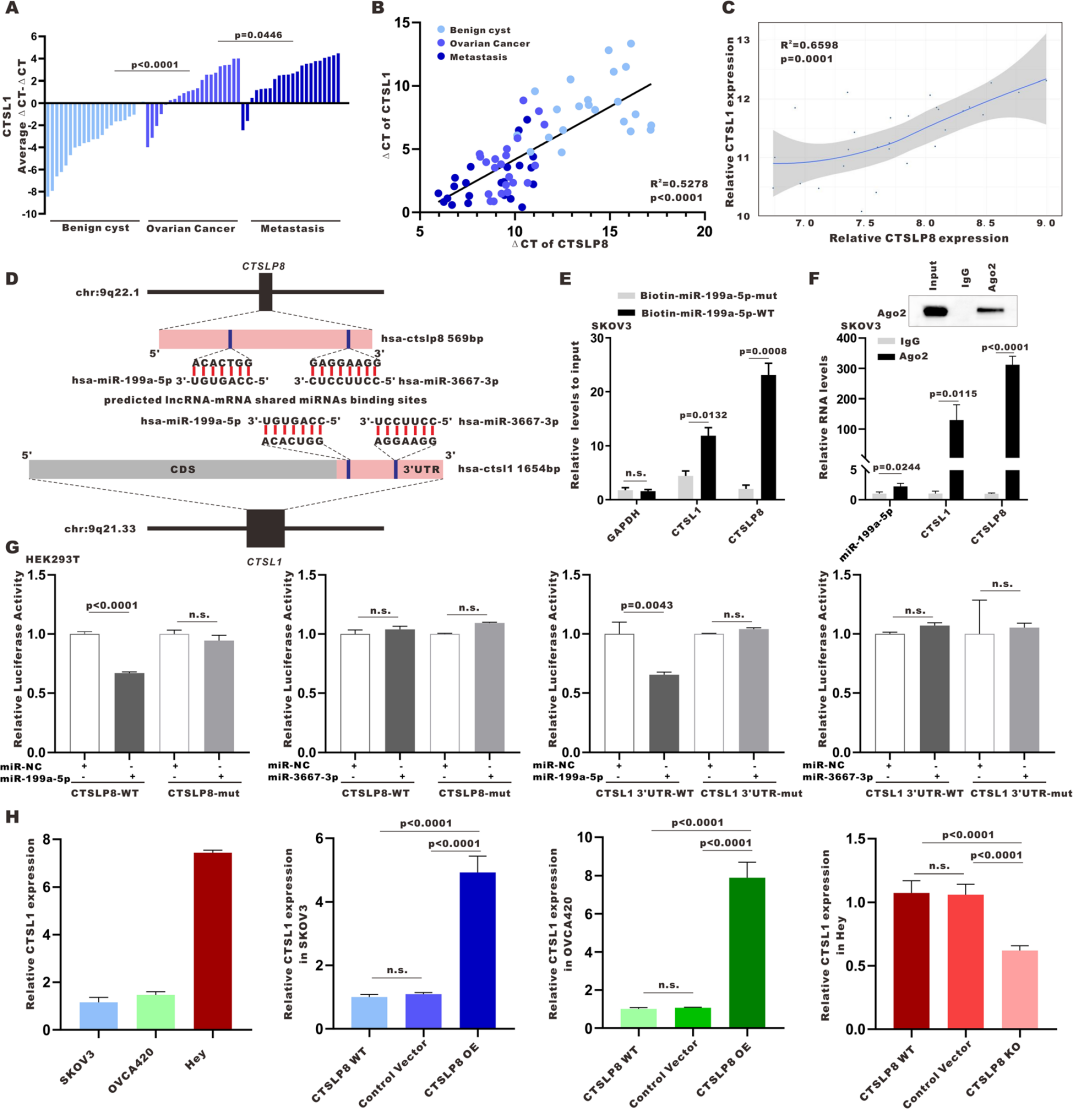
Lnc-CTSLP8 upregulated CTSL1 expression in ovarian cancer by acting as a ceRNA. **a** qRT-PCR of CTSL1 expression in benign ovarian cyst tissues (used as control), ovarian cancer tissues, and matched peritoneal metastasis tissues. **b** The correlation between lnc-CTSLP8 and CTSL1 expression levels in different tissues. **c** GEO data analysis of the correlation between CTSL1 expression and lnc-CTSLP8 expression in ovarian cancer. **d** Prediction of shared miRNAs and miRNA binding sites within lnc-CTSLP8 and CTSL1 3’UTR sequences. **e** Biotinylated wild-type/mutant miR-199a-5p was transfected into SKOV3 cells overexpressing CTSLP8. After streptavidin capture, CTSLP8 and CTSL1 expression were detected via qRT-PCR. **f** RIP experiments were performed using an anti-AGO2 antibody and SKOV3 cells. **g** Luciferase reporter assay using the 3’UTR of CTSL1 and CTSLP8 to analyze repression by the indicated miRNA mimics. Non-specific control miRNA (miR-NC) served as a negative control. **h** qRT-PCR of CTSL1 expression in different cell lines

Using qRT-PCR (Fig. 6a), we found that CTSL1 mRNA expression was also significantly elevated in ovarian cancer tumor tissues (*n* = 22) relative to benign ovarian cyst tissues (*n* = 22) and highest in ovarian cancer peritoneal metastasis tissues (*n* = 22). Furthermore, CTSL1 expression was positively correlated with lnc-CTSLP8 expression in different tissues (Fig. 6b). The same positive correlation was found via GEO ovarian cancer data analysis (Fig. 6c).

We screened miRNA with potential binding sites for both lnc-CTSLP8 and CTSL1–3’UTR. miR-199a-5p and miR-3667-3p were identified using the TargetScan public database (Fig. 6d). To validate the direct binding of miR-199a-5p with both lnc-CTSLP8 and CTSL1, biotin-labeled miR-199a-5p and its mutant mimics were designed to pull down lnc-CTSLP8 and CTSL1 in SKOV3 cells overexpressing lnc-CTSLP8. We found obvious enrichment of lnc-CTSLP8 and CTSL1 for wild-type miR-199a-5p compared with mutant mimics (Fig. 6e). An anti-AGO2 RIP assay was then performed, wherein lnc-CTSLP8, CTSL1, and miR-199a-5p were enriched with AGO2 (Fig. 6f).

Additionally, we tested the predicted shared miRNAs in a luciferase reporter assay and found that miR-199a-5p significantly repressed lnc-CTSLP8 and CTSL1–3’UTR luciferase activity. The mutation of the predicted miRNA binding sites in lnc-CTSLP8 and CTSL1–3’UTR abrogated this effect (Fig. 6g), suggesting that the crosstalk between lnc-CTSLP8 and CTSL1 may be mediated, at least in part, by miR-199a-5p. Moreover, CTSL1 expression was upregulated after lnc-CTSLP8 overexpression and downregulated after lnc-CTSLP8 knockout (Fig. 6h).

The miR-199a-5p expression was significantly decreased in ovarian cancer peritoneal metastasis tissue (Fig. S5A). Further, miR-199a-5p expression exhibited negative correlation with CTSL1 expression (Fig. S5B). Meanwhile, miR-199a-5p expression was significantly altered by the overexpression and knockout of lnc-CTSLP8 (Fig. S5C).

These results indicated that lnc-CTSLP8 may function as a sponge for miR-199a-5p and upregulate CTSL1 as a ceRNA.

### CTSL1 inhibitor reversed the oncogenic effects of lnc-CTSLP8 in vitro and in vivo

**Fig. 7:**
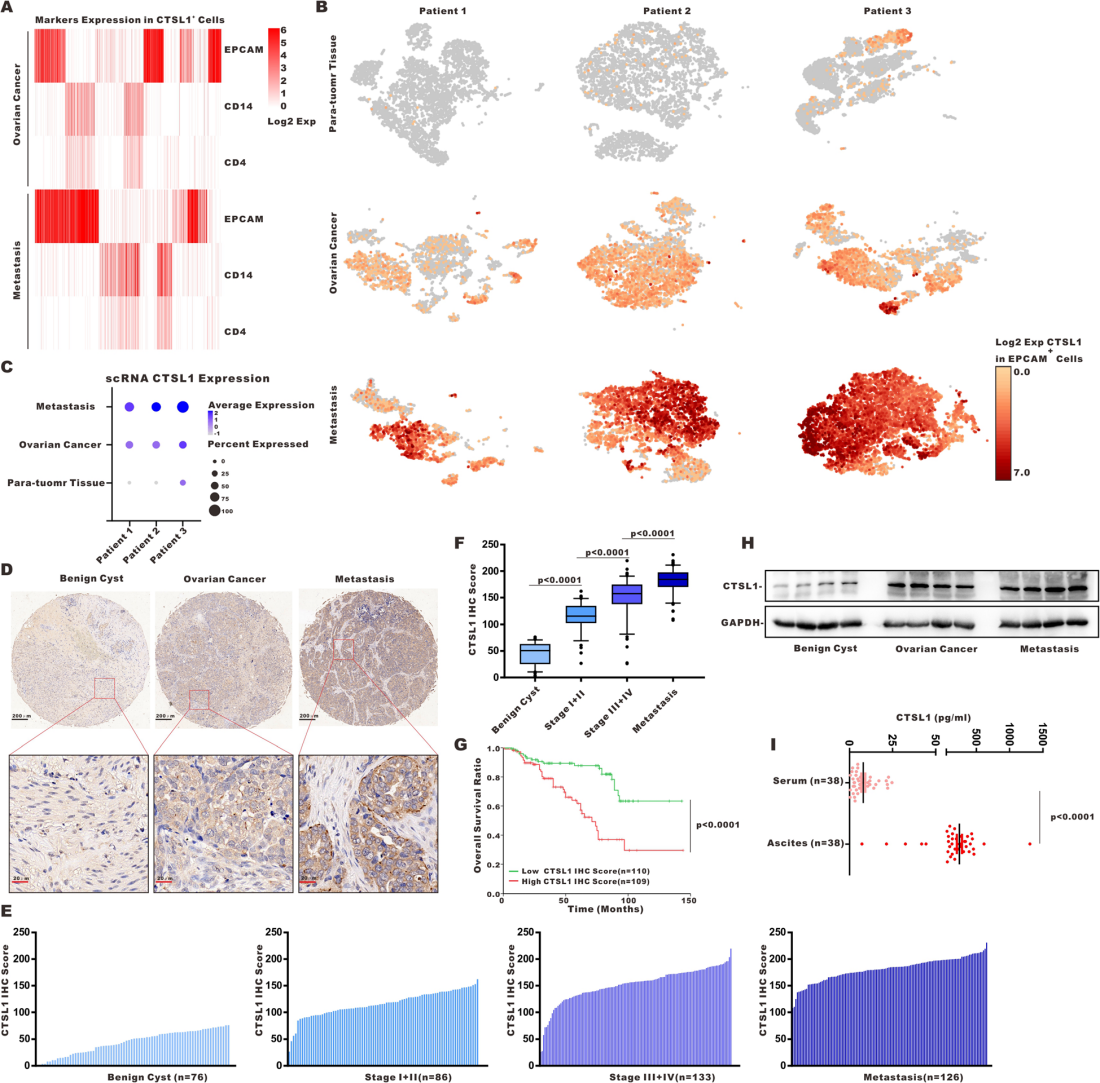
CTSL1 was upregulated in ovarian cancer. **a** Single-cell RNA sequencing (scRNA-seq) analysis of ovarian cancer. The heatmap shows cell marker (EPCAM for epithelial cells, CD14 for monocytes/macrophages, CD4 for CD4^+^ T cells) expression patterns in CTSL1^**+**^ cells. **b** scRNA-seq in EPCAM^**+**^ cells from three ovarian cancer para-tumor tissues (used as control), ovarian cancer tissue, and the matched peritoneal metastasis tissues. **c** Quantification of CTSL1 expression in EPCAM^**+**^ cells from benign ovarian cyst, ovarian cancer, and peritoneal metastasis tissues with different CTSL1 staining. **e** and **f** Quantification of CTSL1 IHC score in different tissues. **g** Overall survival of ovarian cancer patients with low/high CTSL1 IHC score. **h** Immunoblotting of CTSL1 in different tissues. **i** ELISA of CTSL1 in serum and ascites of ovarian cancer patients

We isolated single cells from para-tumor tissues (used as normal controls), ovarian cancer tissues, and peritoneal metastasis tissues from three high-grade serous ovarian cancer patients. Since we barely found CTSL1-positive cells (CTSL1^+^ cells) in para-tumor tissues, the cell markers expression of CTSL1^+^ cells were only analyzed in ovarian cancer and peritoneal metastasis tissues. High EPCAM expression was observed in CTSL1^+^ cells (Fig. 7a), indicating that CTSL1 within the ovarian cancer microenvironment was most likely derived from tumor cells (EPCAM^+^ cells). The expression of monocyte/macrophage marker CD14 and T cell marker CD4 was detected in CTSL1^+^ cells (Fig. 7a). The number of CTSL1^+^ EPCAM^+^ cells and the expression level of CTSL1 in EPCAM^+^ cells were both higher in ovarian cancer tissues than in para-tumor tissues. Further, peritoneal metastasis tissues had the highest number of CTSL1^+^ EPCAM^+^ cells as well as the highest expression level of CTSL1 in these cells (Fig. 7b, c). Similar CTSL1 expression patterns were observed in cells from different tissues (Fig. S6A).

Next, we evaluated CTSL1 protein expression in ovarian cancer tissue. The CTSL1 IHC score of ovarian cancer tissue was significantly higher than that of benign ovarian cyst tissues (Fig. 7d). Moreover, the CTSL1 IHC score increased with disease progression (Fig. 7e, f), and high CTSL1 expression was associated with poor overall survival in ovarian cancer patients (Fig. 7g). The upregulated CTSL1 expression in ovarian cancer and peritoneal metastasis tissues was further confirmed via western blot analysis (Fig. 7h). ELISA revealed that soluble CTSL1 was significantly higher in ascites compared to serum obtained from the same cohort of ovarian cancer patients (Fig. 7i).

**Fig. 8:**
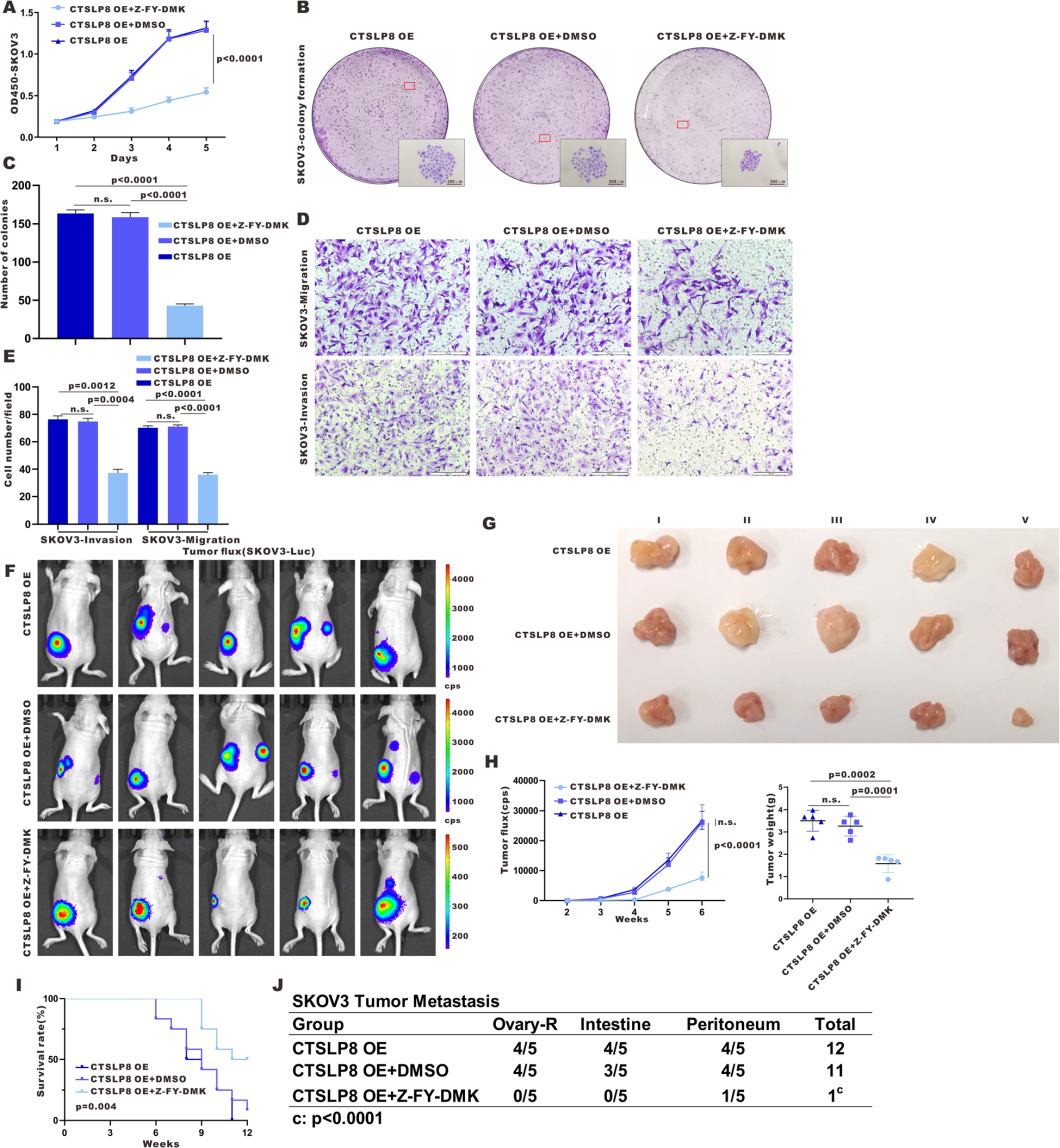
CTSL1 inhibitor treatment reversed the oncogenic effects of lnc-CTSLP8 in vitro and in vivo. **a** Cell viability assay of SKOV3-CTSLP8-OE cell lines treated by CTSL1 inhibitor Z-FY-DMK, DMSO (negative control), or untreated (blank control). **b** and **c** Colony formation in different groups. **d** and **e** The invasion and migration of different groups. **f** Representative bioluminescence image of control and Z-FY-DMK-treated ovarian tumor-bearing mice. **g** Size of ovarian tumors in different mouse groups. **h** Quantification of tumor fluorescence intensity and tumor weight. **i** Survival of tumor-bearing mice in different groups. **j** Metastasis counts in different groups

Next, we inhibited CTSL1 activity in lnc-CTSLP8-overexpressing ovarian cancer cells using Z-FY-DMK, a selective CTSL1 inhibitor. Ectopic expression of lnc-CTSLP8 enhanced the proliferation, migration, and invasion of ovarian cancer cells, but these effects were abrogated by Z-FY-DMK (Fig. 8a-e). Similarly, lnc-CTSLP8 overexpression enhanced tumor formation and tumor weight in vivo, yet these effects were also suppressed in mice treated with Z-FY-DMK (Fig. 8f-g). Further, Z-FY-DMK improved the overall survival of and reduced tumor metastasis in SKOV3-CTSLP8-OE tumor-bearing mice (Fig. 8i,j).

Immunofluorescence assay using the lnc-CTSLP8 probe confirmed that lnc-CTSLP8 overexpression was not affected by Z-FY-DMK treatment (Fig. S7A). The expression of LC3, SQSTM1/p62, E-cadherin, and N-cadherin was then detected via immunofluorescence (Fig. S7B, C). Increased LC3 and SQSTM1/p62 levels were observed in Z-FY-DMK-treated SKOV3-CTSLP8-OE ovarian tumor tissues. Further, the EMT process was inhibited by Z-FY-DMK.

### CTSL1 inhibitor and miR-199a-5p overexpression abrogated lnc-CTSLP8-promoted EMT and autophagy in ovarian cancer

**Fig. 9:**
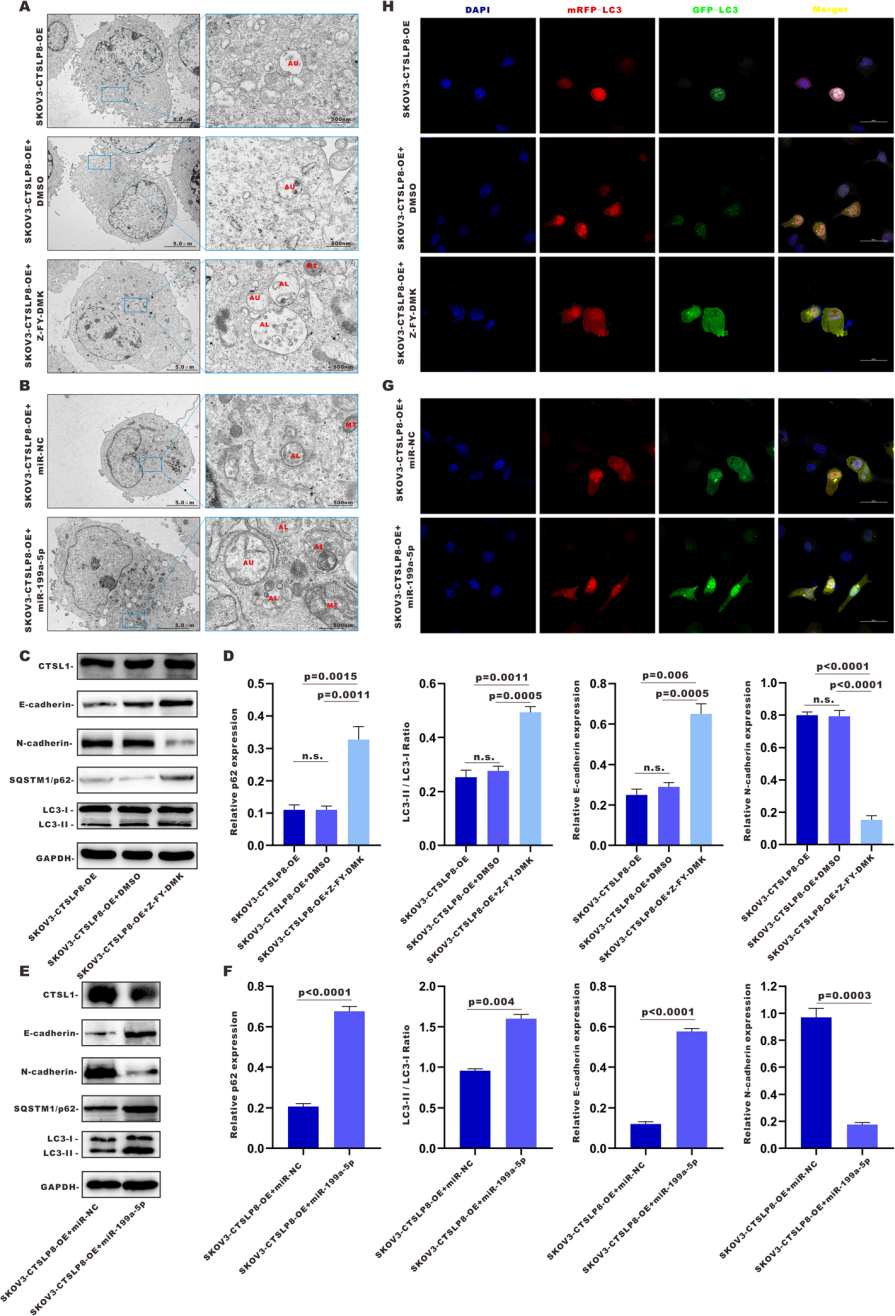
CTSL1 inhibitor and miR-199a-5p abrogated the lnc-CTSLP8-mediated promotion of EMT and autophagy in ovarian cancer. **a** TEM of the AU, AL, and MT in SKOV3-CTSLP8-OE cells treated with CTSL1 inhibitor Z-FY-DMK, DMSO (negative control), or untreated (blank control). **b** TEM of the AU, AL, and MT in SKOV3-CTSLP8-OE cells treated with miR-199a-5p or miR-NC (negative control). **c** and **e** Immunoblotting of autophagy-related genes and EMT markers in different cell lines. **d** and **f** Quantification of relative p62 expression, LC3-II/ LC3-I ratio, and EMT marker expression in different cell lines. **h** and **f** Immunofluorescence assay of mRFP-GFP-LC3 in different cell lines

The number of enlarged autophagosomes (AU), autolysosomes (AL), and dysfunctional mitochondria (MT) increased in SKOV3-CTSLP8-OE cells treated with Z-FY-DMK, but not in their DMSO-treated counterparts (Fig. 9a). Z-FY-DMK treatment elevated SQSTM1/p62 expression and the LC3-II/LC3-I ratio in SKOV3-CTSLP8-OE cells (Fig. 9c, d), indicating that the promotion of autophagy by lnc-CTSLP8 overexpression was reversed by the CTSL1 inhibitor. The blockade of autophagy flux by Z-FY-DMK was then confirmed via mRFP-GFP-LC3 immunofluorescence (Fig. 9h).

As an CTSL1-targeting miRNA, miR-199a-5p mediates ceRNA regulation between lnc-CTSLP8 and CTSL1. Therefore, miR-199a-5p was transfected into SKOV3-CTSLP8-OE cells to confirm its regulatory role. TEM analyses revealed that miR-199a-5p increased the number of enlarged AU, AL, and dysfunctional mitochondria MT in SKOV3-CTSLP8-OE cells relative to miR-NC (Fig. 9b). Immunoblotting analyses indicated that CTSL1 expression was decreased by miR-199a-5p. The SQSTM1/p62 expression and LC3-II/LC3-I ratio were elevated in SKOV3-CTSLP8-OE cells (Fig. 9e, f), suggesting that the promotion of autophagy by lnc-CTSLP8 overexpression should be reversed by miR-199a-5p. The blockade of autophagy flux by miR-199a-5p was then confirmed via mRFP-GFP-LC3 immunofluorescence detection (Fig. 9g). Immunofluorescence assays of LC3 in SKOV3-CTSLP8-OE cells treated with Z-FY-DMK or miR-199a-5p were conducted, revealing that LC3 expression increased after Z-FY-DMK or miR-199a-5p treatment (Fig. S8A, D).

Furthermore, the EMT promoted by lnc-CTSLP8 overexpression was also abrogated in cells treated with Z-FY-DMK or miR-199a-5p, since the expression of E-cadherin was significantly increased, while that of N-cadherin was significantly reduced, as determined via immunoblotting (Fig. 9c, f). Immunofluorescence assays of E-cadherin and N-cadherin confirmed these results (Fig. S8C, F). In addition, the expression of ZEB1 and Snail, key regulators of the EMT, was detected by immunofluorescence staining (Fig. S8B, E), showing that Z-FY-DMK and miR-199a-5p reduced the expression of both regulators in SKOV3-CTSLP8-OE cells.

## Discussion

Pseudogenes are involved in human malignancies through their function as ceRNAs for their cognate genes due to sharing the same miRNA response elements [23]. Whether pseudogenes function as tumor suppressors or oncogenes depends on their protein-coding gene counterparts. For instance, pseudogene ceRNA networks of BRAFP1-BRAF [17] and KRASP1-KRAS [24] were shown to promote tumorigenesis. In this study, we investigated the role of pseudogenes in ovarian cancer, with a particular focus on CTSL pseudogene 8, as qRT-PCR confirmed its upregulation in metastatic ovarian cancer tissues. Further, public database analysis also revealed lnc-CTSLP8 upregulation in advanced-stage or high-grade ovarian cancer patients. Through overexpression/knockout of lnc-CTSLP8 in ovarian cancer cells in vitro and in vivo, we established lnc-CTSLP8 as a potent oncogene, which acts by enhancing the autophagy and EMT in ovarian cancer cells.

Mechanistically, lnc-CTSLP8 contributed to the progression of ovarian cancer by acting as a ceRNA to its cognate gene, CTSL1. Following previous models of ceRNA interactions [25, 26], we confirmed the regulation between lnc-CTSLP8 and CTSL1 via miRNA binding prediction, RIP, and target site mutation analyses.

CTSL1, a family member of cysteine cathepsins, is a crucial lysosomal enzyme. Tumor cell-derived CTSL1 was reported to have a tumor-promoting function, as the CTSL1 knockout phenotype in mice could not be rescued via transplantation of wild-type donor bone marrow [27]. In this study, we confirmed that ovarian cancer cells were the major source of CTSL1, and soluble CTSL1 was enriched in malignant ascites fluid. CTSL1 deficiency could lead to defects in autophagy, characterized by large dysmorphic vesicles and the accumulation of mitochondria within the cytoplasm [28]. Moreover, defects in autophagy could also be characterized by an increased LC3-II/LC3-I ratio and the accumulation of the autophagy substrate SQSTM1/p62 [29]. We observed these autophagy defects in lnc-CTSLP8 knockout ovarian cancer cells. Secreted CTSL1 was reported to promote cancer cell invasion and EMT by cleaving cell adhesion molecule E-cadherin [19]. As expected, we detected increased N-cadherin and reduced E-cadherin in lnc-CTSLP8 OE ovarian expression. Surprisingly, EMT-related transcription factors ZEB1 and Snail were also regulated by lnc-CTSLP8, and further studies are needed to elucidate the underlying mechanisms.

CTSL1 is dysregulated in various human diseases and represents a promising drug target [30, 31]. In this study, CTSL1 inhibition abrogated the oncogenic effects of lnc-CTSLP8. In particular, the enhancement of autophagy and EMT observed in lnc-CTSLP8 OE cancer cells was reversed by treatment with a selective CTSL1 inhibitor, indicating that the function of lnc-CTSLP8 depended on CTSL1 activity. Whether the oncogenic effects of lnc-CTSLP8 involve other ceRNA targets remains to be addressed in further research.

## Conclusion

In summary, lnc-CTSLP8 upregulates CTSL1 by acting as a decoy against their shared miRNA, miR-199a-5p. Consequently, the pseudogene-gene functional network promotes ovarian cancer metastasis via the induction of EMT and autophagy in cancer cells. Moreover, the oncogenic effects of lnc-CTSLP8 can be abrogated by CTSL1 selective inhibitor treatment. Collectively, our study reveals a novel ceRNA mechanism and identifies lnc-CTSLP8 as a potential therapeutic target for metastatic ovarian cancer.

## Supplementary Information

**Additional file 1: Supplementary Table 1.** The clinicopathological characteristics and overall survival in ovarian cancer patients with different CTSL1 expression.

**Additional file 2: Supplementary Table 2.** sgRNA sequence.

**Additional file 3: Supplementary Table 3**. Primer sequence.

**Additional file 4: Supplementary Table 4.** Primary antibodies.

**Additional file 5: Figure S1.** CTSLP8 overexpression and knockout cell lines were constructed for further study. (A) Immunofluorescence assay using an RNA probe designed for CTSLP8 (Red) in CTSLP8 overexpression and knockout cell lines. (B) sgRNAs designed for CTSLP8 knockout. (C) Structure of the two-gRNA-expressing plasmid. (D) Sanger DNA sequencing of knockout cell line.

**Additional file 6: Figure S2.** CTSLP8 overexpression promoted ovarian cancer progression in vitro and in vivo. (A) Viability assay of OVCA420-CTSLP8-OE cells, OVCA420 cells transfected with a control vector (negative control), and wild-type OVCA420 (blank control). Significance was calculated via the two-way ANOVA test. (B and C) The colony formation of different groups (Student’s t-test). (D and E) The invasion and migration of different groups (Student’s t-test). (F) Representative bioluminescence image of control and CTSLP8-OE OVCA420 ovarian tumor-bearing mice. (G) Size of ovarian tumors in different mouse groups. (H) Quantification of tumor fluorescence intensity (two-way ANOVA test) and tumor weight (Student’s t-test). (I) Tumor-bearing mouse survival in different groups (Kaplan–Meier survival analysis). (J) Metastasis counts in different groups (chi-square test).

**Additional file 7: Figure S3.** Lnc-CTSLP8 regulated LC3 and EMT-related transcription factors in ovarian cancer cells. (A) Immunofluorescence assay of LC3 (red) in different cell lines. (B) Immunofluorescence assay of EMT-related transcription factors ZEB1 (red) and Snail (green) in different cell lines.

**Additional file 8: Figure S4.** Lnc-CTSLP8 promoted EMT and autophagy in ovarian cancer in vivo. (A) Immunofluorescence assay using an RNA probe design for CTSLP8 (red) in ovarian cancer tissues of tumor-bearing mice. (B) Immunofluorescence assay for LC3 (red) and p62 (green) in ovarian cancer tissues of tumor-bearing mice.

**Additional file 9: Figure S5.** miR-199a-5p was negatively correlated with CTSL1 expression in ovarian cancer. (A) qRT-PCR of relative miR-199a-5p expression in benign ovarian cyst tissues (used as control), ovarian cancer tissues, and matched peritoneal metastasis tissues (Student’s t-test). (B) Correlation analysis between miR-199a-5p and CTSL1 expression levels in different tissues (linear regression analysis). (C) qRT-PCR of relative CTSL1 expression in different cell lines (Student’s t-test).

**Additional file 10: Figure S6.** CTSL1 expression was significantly elevated in ovarian cancer tissues and metastasis tissues. (A) scRNA-seq in all cells from three ovarian cancer para-tumor tissues (used as control), ovarian cancer tissues, and the matched peritoneal metastasis tissues.

**Additional file 11: Figure S7.** CTSL1 inhibition reversed the CTSLP8-mediated promotion of EMT and autophagy in ovarian cancer in vivo. (A) Immunofluorescence assay using an RNA probe against CTSLP8 (red) in ovarian cancer tissues of tumor-bearing mice. (B) Immunofluorescence assay for LC3 (red) and p62 (green) in ovarian cancer tissues of tumor-bearing mice.

**Additional file 12: Figure S8.** CTSL1 inhibition and miR-199a-5p reversed the CTSLP8-mediated promotion of EMT and autophagy in ovarian cancer cells. (A and D) Immunofluorescence assay of LC3 (red) in different cell lines. (B and E) Immunofluorescence assay of EMT-related transcription factors ZEB1 (red) and Snail (green) in different cell lines. (C and F) Immunoblotting of EMT markers in different cell lines.

## Data Availability

All data used or analyzed during this study are included in this article and are available from the corresponding author upon reasonable request.

## Abbreviations

lncRNA: Long non-coding RNA
CTSLP8: Cysteine cathepsin L pseudogene 8
CTSL1: Cysteine cathepsin L 1
ceRNA: Competing endogenous RNA
PTEN: Phosphatase and tensin homolog
BRAF: B-Raf proto-oncogene, serine/threonine kinase
KRAS: KRAS proto-oncogene, GTPase
TCGA: The Cancer Genome Atlas
GEO: Gene Expression Omnibus
ZEB1: Zinc finger E-box binding homeobox 1
LC3: Microtubule associated protein 1 light chain 3 alpha
EMT: Epithelial-mesenchymal transition
EPCAM: Epithelial cell adhesion molecule
WT: Wild type
OE: Overexpression
KO: Knock out
NC: Negative control
