# Supplementary material for: The lnc-CTSLP8 upregulates CTSL1 as a competitive endogenous RNA and promotes ovarian cancer metastasis
Source: J Exp Clin Cancer Res. 2021 May 1;40:151. doi: 10.1186/s13046-021-01957-z (PMC8088648; doi:10.1186/s13046-021-01957-z)
Supplement: Supplementary file 1 — Additional file 1: Supplementary Table 1. The clinicopathological characteristics and overall survival in ovarian cancer patients with different CTSL1 expression. [file 13046_2021_1957_MOESM1_ESM.docx]

**Supplementary Table 1.** **The clinicopathological characteristics and overall survival in ovarian cancer patients with different CTSL1 expression.**

| **Characteristics** |  | **Low CTSL1（n=110）** | | **High CTSL1 （n=109）** | | **P value** |
| --- | --- | --- | --- | --- | --- | --- |
| Patients age | Range | 26-81 | 33-85 | | 0.1933 | |
|  | Mean ± SD | 55.99±8.98 | 54.41±8.92 | |  |  |
| FIGO stage | I+II | 72 | 14 | | <0.0001 | |
|  | III+IV | 38 | 95 | |  |  |
| Overall survival (months) | Range | 2-143 | 1-144 | | 0.0025 | |
|  | Mean ± SD | 56.45±34.88 | 43.7±26.17 | |  |  |

Student t test and Chi-square test.
