# Supplementary material for: The lnc-CTSLP8 upregulates CTSL1 as a competitive endogenous RNA and promotes ovarian cancer metastasis
Source: J Exp Clin Cancer Res. 2021 May 1;40:151. doi: 10.1186/s13046-021-01957-z (PMC8088648; doi:10.1186/s13046-021-01957-z)
Supplement: Supplementary file 2 — Additional file 2: Supplementary Table 2. sgRNA sequence. [file 13046_2021_1957_MOESM2_ESM.docx]

**Supplementary Table 2.** **sgRNA sequence**

| **sgRNA** | **sequence** |
| --- | --- |
| **sgRNA1** | **AGGATGGAGGAGAGCAGTGT** |
| **sgRNA2** | **TTGTGAAGCTGTGTTTCCCT** |
| **sgRNA3** | **GAGACATGGTGAGTGTGCTG** |
| **sgRNA4** | **TACAAAAGAAAGAGGTTTAA** |
| **sgRNA5** | **ACTCACAGTTCCACATGGCA** |
| **sgRNA6** | **ATGAGGCCTCCCCTGCCATG** |
| **sgRNA7** | **GAAAATGAGCGTCAAGCGAA** |
| **sgRNA8** | **CATTATCATGAGAACAGCAT** |
