# Supplementary material for: The lnc-CTSLP8 upregulates CTSL1 as a competitive endogenous RNA and promotes ovarian cancer metastasis
Source: J Exp Clin Cancer Res. 2021 May 1;40:151. doi: 10.1186/s13046-021-01957-z (PMC8088648; doi:10.1186/s13046-021-01957-z)
Supplement: Supplementary file 3 — Additional file 3: Supplementary Table 3. Primer sequence. [file 13046_2021_1957_MOESM3_ESM.docx]

**Supplementary Table 3.** **Primer sequence**

| **Gene** | **Primer sequence** | **Application** |
| --- | --- | --- |
| **CTSLP8** | **Forward Primer: CCATCTCTGTTGCTGTTG** | **qRT-PCR &** |
|  | **Reverse Primer: TCCTTCCTCATCACCATC** | **RNA pull down** |
| **CTSL1** | **Forward Primer: CTTTTGCCTGGGAATTGCCTC** | **qRT-PCR &** |
|  | **Reverse Primer: CATCGCCTTCCACTTGGTC** | **RNA pull down** |
| **GAPDH** | **Forward Primer: GGAGCGAGATCCCTCCAAAAT** | **qRT-PCR &** |
|  | **Reverse Primer: GGCTGTTGTCATACTTCTCATGG** | **RNA pull down** |
| **CTSLP8** | **Forward Primer: cggaggaacaactgtggaat** | **RIP** |
|  | **Reverse Primer: tcgagtccttcctcatcacc** |  |
| **CTSL1** | **Forward Primer: agaccggagaaaccattgtg** | **RIP** |
|  | **Reverse Primer: cccagtcaagtccttcctca** |  |
| **miR-199a-5p** | **Forward Primer: gtggtcccagtgttcagacta** | **RIP** |
|  | **stem-loop: attcgcaccagagccaacgaacag** |  |
