# Supplementary material for: The lnc-CTSLP8 upregulates CTSL1 as a competitive endogenous RNA and promotes ovarian cancer metastasis
Source: J Exp Clin Cancer Res. 2021 May 1;40:151. doi: 10.1186/s13046-021-01957-z (PMC8088648; doi:10.1186/s13046-021-01957-z)
Supplement: Supplementary file 4 — Additional file 4: Supplementary Table 4. Primary antibodies. [file 13046_2021_1957_MOESM4_ESM.docx]

**Supplementary Table 4. Primary antibodies**

| **Primary antibodies** | **SOURCE** | **IDENTIFIER** | **Application** |  |
| --- | --- | --- | --- | --- |
| **CTSL1** | **Abcam** | **Cat# ab58991** | **IF** |  |
| **CTSL1** | **R&D Systems** | **Cat# MAB952** | **WB** |  |
| **E-Cadherin** | **Cell Signaling Technology** | **Cat #3195** | **WB&IF** |  |
| **N-cadherin** | **Cell Signaling Technology** | **Cat #13116** | **WB** |  |
| **N-cadherin** | **Proteintech** | **Cat# 66219-1-Ig** | **IF** |  |
| **Snail** | **Cell Signaling Technology** | **Cat #3879** | **WB** |  |
| **Snail** | **Proteintech** | **Cat# 13099-1-Ap** | **IF** |  |
| **ZEB1** | **Cell Signaling Technology** | **Cat #3396** | **WB** |  |
| **ZEB1** | **Abcam** | **Cat# ab181451** | **IF** |  |
| **ATG3** | **Cell Signaling Technology** | **Cat# 3415** | **WB** |  |
| **ATG7** | **Cell Signaling Technology** | **Cat# 8558** | **WB** |  |
| **ATG12** | **Cell Signaling Technology** | **Cat# 2010** | **WB** |  |
| **LC3** | **Cell Signaling Technology** | **Cat# 4108** | **WB&IF** |  |
| **SQSTM/p62** | **Cell Signaling Technology** | **Cat# 88588** | **WB&IF** |  |
