# Supplementary figures and images for: The lnc-CTSLP8 upregulates CTSL1 as a competitive endogenous RNA and promotes ovarian cancer metastasis
Source: J Exp Clin Cancer Res. 2021 May 1;40:151. doi: 10.1186/s13046-021-01957-z (PMC8088648; doi:10.1186/s13046-021-01957-z)

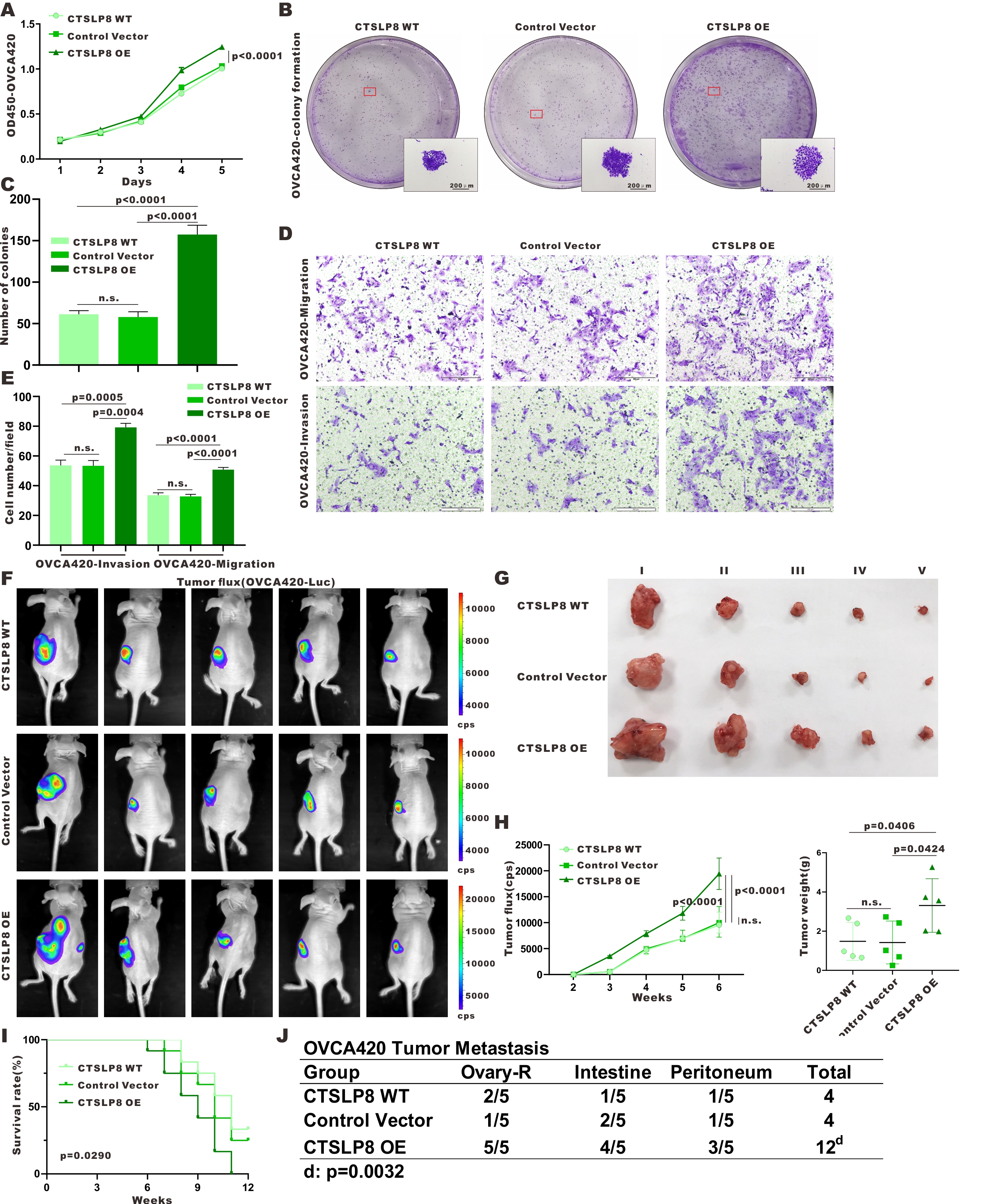

Supplement: Supplementary file 6 — Additional file 6: Figure S2. CTSLP8 overexpression promoted ovarian cancer progression in vitro and in vivo. (A) Viability assay of OVCA420-CTSLP8-OE cells, OVCA420 cells transfected with a control vector (negative control), and wild-type OVCA420 (blank control). Significance was calculated via the two-way ANOVA test. (B and C) The colony formation of different groups (Student’s t-test). (D and E) The invasion and migration of different groups (Student’s t-test). (F) Representative bioluminescence image of control and CTSLP8-OE OVCA420 ovarian tumor-bearing mice. (G) Size of ovarian tumors in different mouse groups. (H) Quantification of tumor fluorescence intensity (two-way ANOVA test) and tumor weight (Student’s t-test). (I) Tumor-bearing mouse survival in different groups (Kaplan–Meier survival analysis). (J) Metastasis counts in different groups (chi-square test). [file 13046_2021_1957_MOESM6_ESM.jpg]

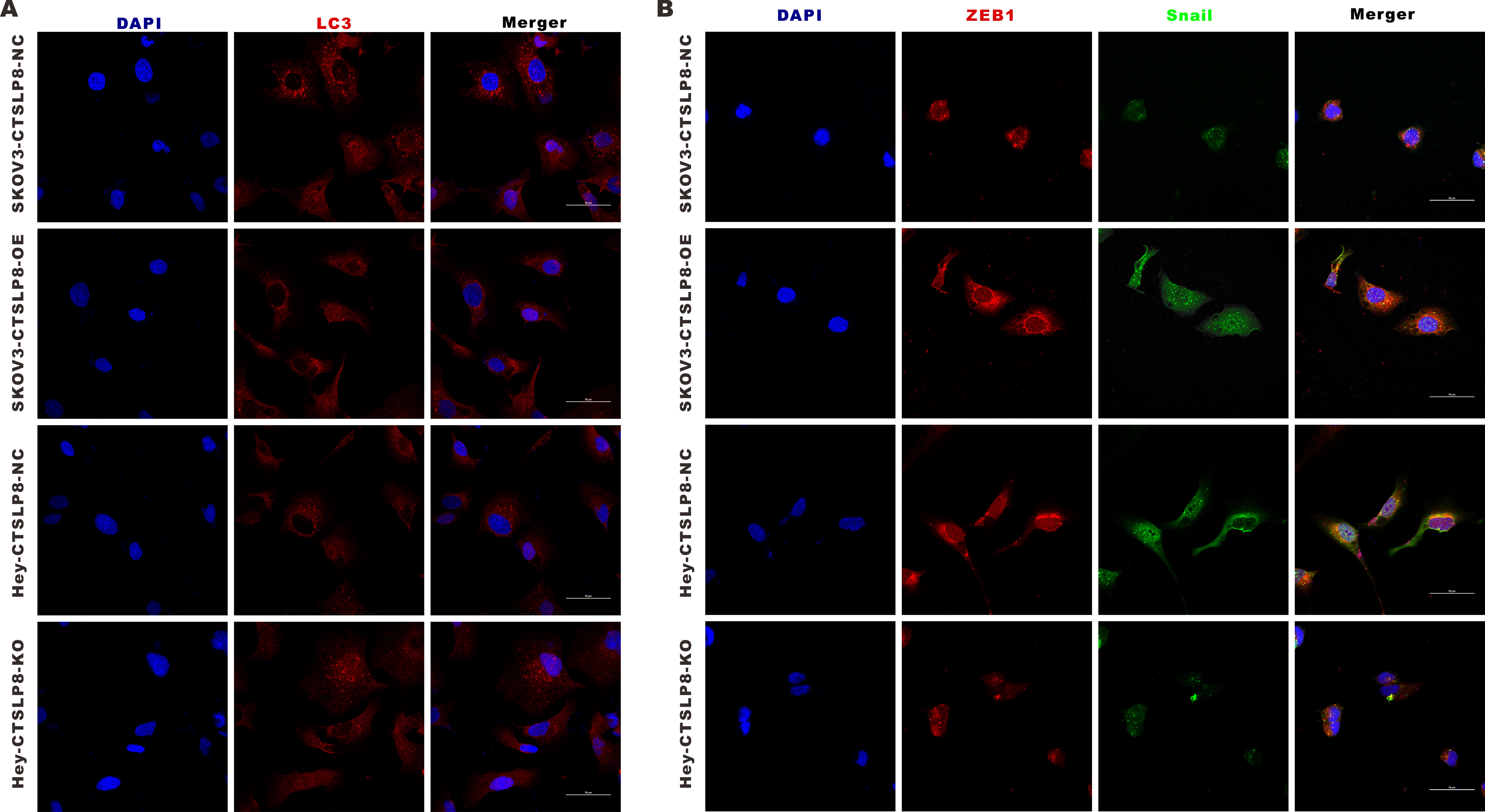

Supplement: Supplementary file 7 — Additional file 7: Figure S3. Lnc-CTSLP8 regulated LC3 and EMT-related transcription factors in ovarian cancer cells. (A) Immunofluorescence assay of LC3 (red) in different cell lines. (B) Immunofluorescence assay of EMT-related transcription factors ZEB1 (red) and Snail (green) in different cell lines. [file 13046_2021_1957_MOESM7_ESM.jpg]

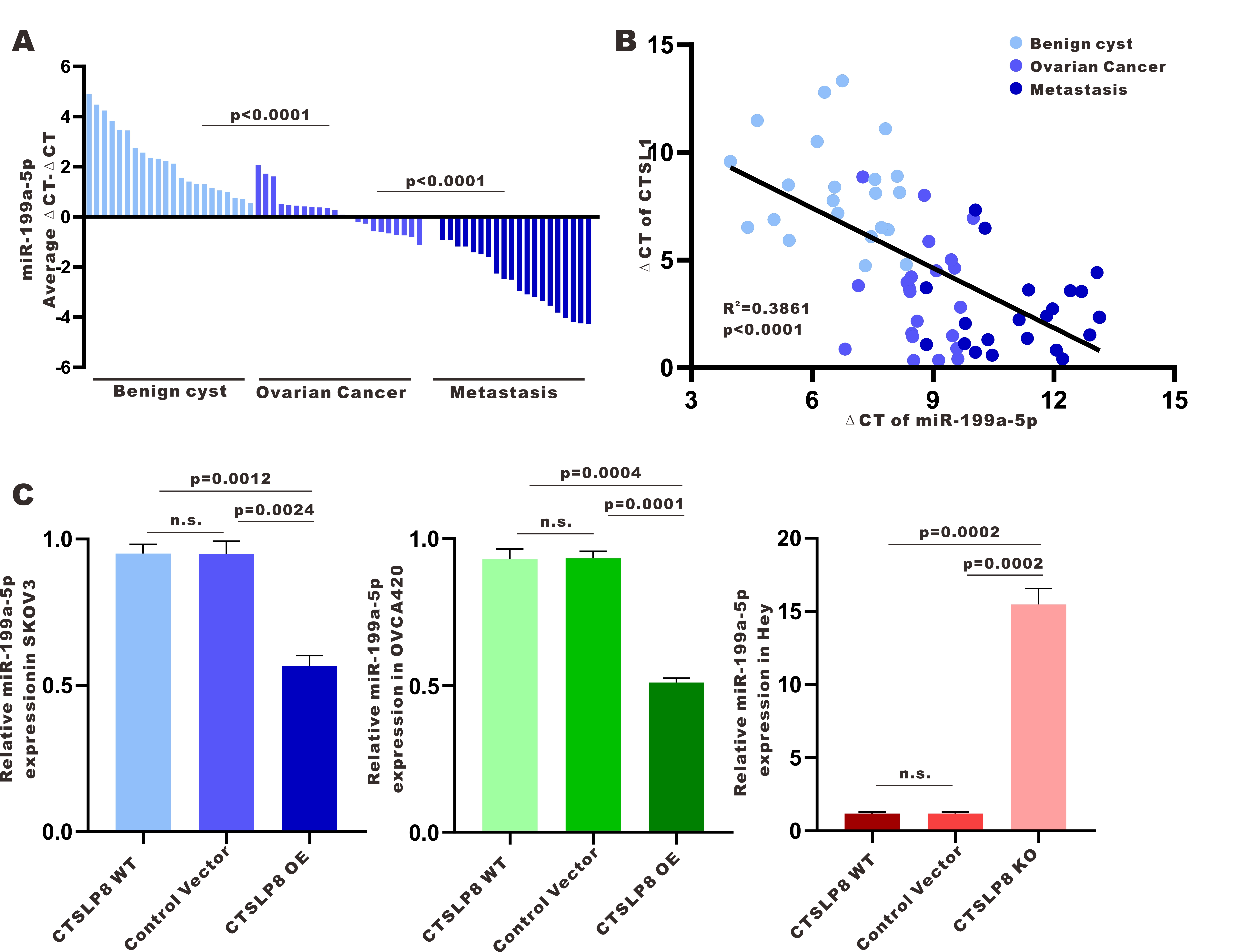

Supplement: Supplementary file 9 — Additional file 9: Figure S5. miR-199a-5p was negatively correlated with CTSL1 expression in ovarian cancer. (A) qRT-PCR of relative miR-199a-5p expression in benign ovarian cyst tissues (used as control), ovarian cancer tissues, and matched peritoneal metastasis tissues (Student’s t-test). (B) Correlation analysis between miR-199a-5p and CTSL1 expression levels in different tissues (linear regression analysis). (C) qRT-PCR of relative CTSL1 expression in different cell lines (Student’s t-test). [file 13046_2021_1957_MOESM9_ESM.jpg]

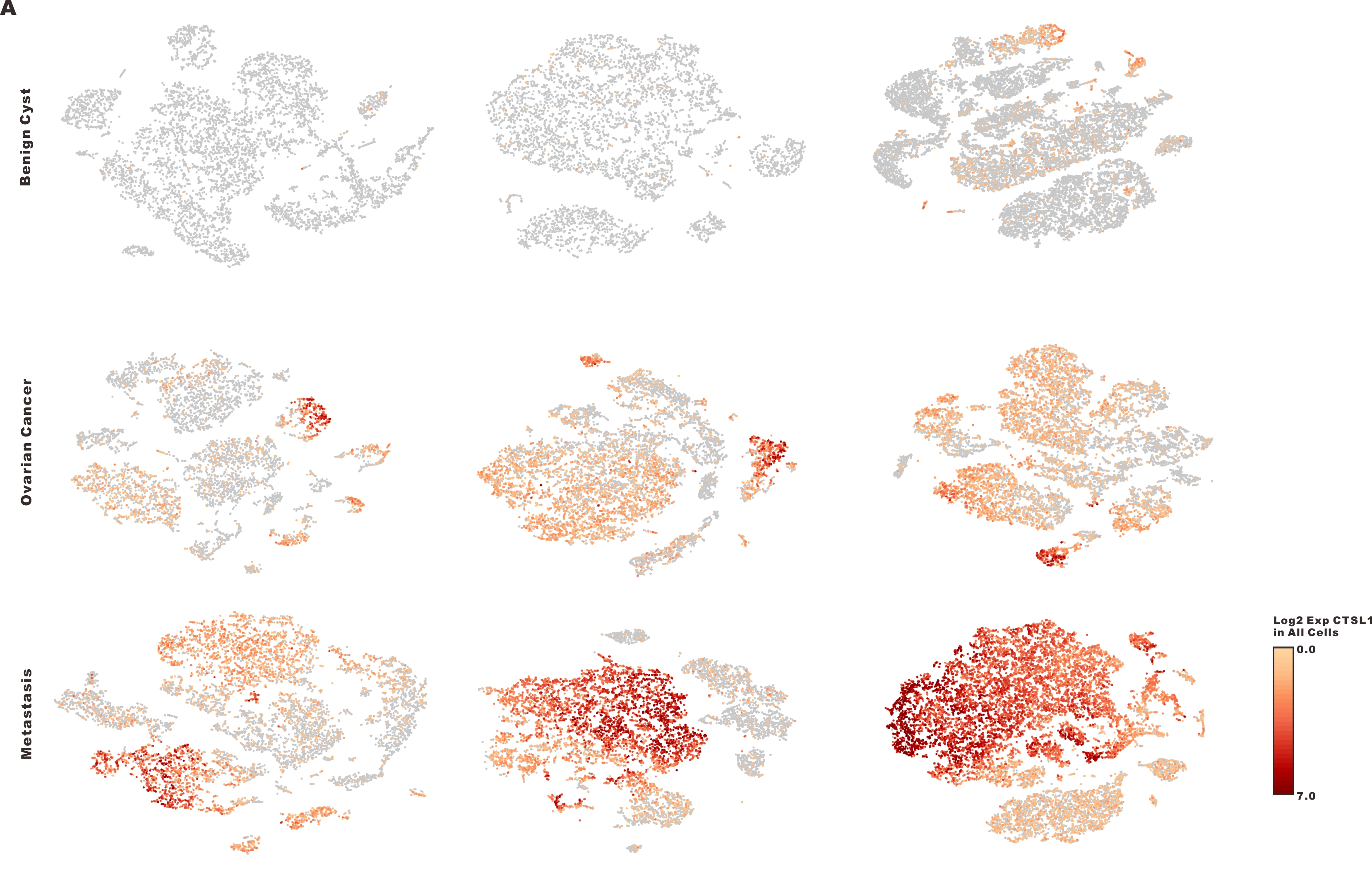

Supplement: Supplementary file 10 — Additional file 10: Figure S6. CTSL1 expression was significantly elevated in ovarian cancer tissues and metastasis tissues. (A) scRNA-seq in all cells from three ovarian cancer para-tumor tissues (used as control), ovarian cancer tissues, and the matched peritoneal metastasis tissues. [file 13046_2021_1957_MOESM10_ESM.jpg]
